# The East Tennessee Record of Medicine and Surgery

**Published:** 1852-06

**Authors:** 


*The East Tennessee Record of Medicine and Surgery.* Edited by Frank A. Ramsay, A.M., M.D. For April, 1852.

This is a quarterly, each No. containing 100 pages octavo, published at Knoxville, under the auspices of the East Tennessee Medical Society. We have placed it on our list of exchanges.

